# Medicine and Surgery

**Published:** 1920-12

**Authors:** L. E. V. Every-Clayton


*"Scire est nescire, nisi id me
Scire alius sciret."*

DECEMBER, 1920.

### MEDICINE AND SURGERY.

The Presidential Address, delivered on October 13th, 1920, at the opening of the Forty-seventh Session of the Bristol Medico-Chirurgical Society.

BY

L. E. V. EVERY-CLAYTON, M.D. Lond.

THIS Society, which has honoured me by electing me its President, is called the Bristol Medico-Chirurgical Society. The name implies that it concerns itself with both of the great branches of special knowledge with which we doctors have to do. I take as the subject of my address, "Medicine and Surgery." The term medicine has at least three meanings. The first finds expression in the vernacular as "a bottle of stuff"; the second regards medicine as identical with the healing art; while in the third connotation medicine is recognised in her true place as the part of a whole, the whole being science, which is organised knowledge of Nature, the part medicine being organised knowledge of disease. But what is the meaning of disease? In the September number of *The Practitioner* Sir Clifford Allbutt tries his by no means 'prentice hand at the framing of a definition. "A disease," says that acknowledged leader of medicine, "seems to be a series of events, concurrent and consecutive, that tends to recur with fair uniformity." The same remark might, with equal propriety, be made about the weather. Sir Frederick Taylor would have the student know that "a disease is any divergence from health;" while Cohnheim takes us a step farther with this oracle, "Disease, then, is a condition in which the regulative mechanisms of the body, acting in opposition to one or more external influences, are no longer adequate to secure that the various physiological functions shall proceed undisturbed."

The best indication of the right use of the word disease that I have yet met with is that of the late Dr. Sydney Macilwaine, who spent the last years of his life in retirement in Clevedon, and who in the leisure thus afforded him wrote a little book entitled *Medical Revolution*, in which this definition appears: "Disease," says Dr. Macilwaine, "is a conception drawn from the observation of a series of cases presenting symptom-groups of determinate and similar causation." I would have you note that "a conception," an action of the mind, not a substance nor an entity. This definition of disease is positive, not negative; moreover, it is clear, practical, and helpful. A symptom-group, or syndrome as we sometimes like to call it, gains recognition by repeated clinical observation. Presently, clinical or experimental observation, *post-mortem* findings, and the bacteriological laboratory—one or other of these—gives us the cause. The whole process repeats itself again and again, and a conception is formed of symptom-group plus constant cause, which is a disease.

The next word of most frequent use in our vocabulary is "diagnosis," and here again I find myself unable to improve upon the connotation which Dr. Macilwaine ascribes to it. Diagnosis finds its proper sphere of action in

"the correlation of cause and effect in the individual patient." A case may present the well-known symptom-complex of intense headache, giddiness and vomiting, dimness of sight, and epileptiform convulsions. It is only when we are able to ascribe these signs and symptoms to the presence of a tumour of the brain that we have made a diagnosis and named a disease.

At the Cambridge meeting of the British Medical Association the subject of Medical Education took a prominent place. Each professor of a subject constituted himself a special pleader, and succeeded admirably in making out a case for the inclusion, as the case might be, of Chemistry, Physics, Biology, Anatomy, in the medical curriculum. The idea of dividing up scientific subjects into water-tight compartments is, to my thinking, fundamentally wrong, and I wish to protest here emphatically against it. All science is one. The unity of science must be an article in our creed. How can it be otherwise if we grant the truth of the definition that science is organised knowledge of Nature, *i.e.* the study and investigation of the structure, substance, and inter-relation of that natural universe in which man finds himself, and of which he is a part? It is cosmos, not chaos, with which we have to do, and it is the existence of design, order and law which makes science possible. No science stands entirely alone, or can afford to ignore, as with a sort of social superiority, the existence of other sciences, and there is no science in relation to which medicine can say, "I have no need of you." I do not think we can abbreviate the curriculum by discarding subjects, but by modification and limitation in the way those subjects are taught. Rather than abbreviate the curriculum, I would add to the list of subjects that of Medical History, the story of knowledge and its acquirement. Indeed, it were well if the various branches of science were expounded in a more historical manner. I

well remember, in my own student days, how greatly the difficulty of understanding the then accepted theory of the coagulation of the blood was increased by not knowing how the present facts, or supposed facts, had been arrived at.

Sir James Mackenzie, in his new venture at St. Andrew's, has focused attention on our need of still more detailed and still more accurate collecting and sifting of the facts gained by clinical observation. It is in this exact, precise, and complete observation of symptoms and signs, and in the correlation of these with morbidity of structure or function, or both, that the general practitioner can find his true opportunity of contributing to the sum-total of our knowledge. It should be a golden rule of practice always to try to find an answer to the question, What is the meaning of each and every sign and symptom which may be present in any given case? and to make particular note of any to which the answer is not immediately forthcoming. It is impossible, with the uncertainty of the calls upon his time, that the busy practitioner should do research work along laboratory lines, but in the field of precise clinical observation he will always find work to his hand, and I entirely agree with Dr. Lewis when he points out (in his recent lecture on the relation of physiology to medicine) that the main function of cumbrous, elaborate, and expensive apparatus is to establish facts which must thereafter be correlated with clinical practice.

We have already made the acquaintance of medicine as mistress of the healing art, and medicine as the science of disease; we must not entirely forget medicine in her more humble rôle as the actual substance used in the treatment of the sick. Her name is then Materia Medica, and I think she has fallen upon evil days. I sometimes wonder whether the profession is aware how much it is drifting into the hands of the big commercial houses and manufacturing chemists, both in the matter of its therapeutics, its choice of remedies, and in the matter of a somewhat hazy knowledge of what those remedies are and can do. Practitioners must regain their lost control in this business, and see to it that all drugs come into their hands with trustworthy scientific credentials.

Sir George Newman, in his extremely able and exhaustive memorandum, has approached the vital question of preventive medicine from the point of view of the administrator and sociologist. The aspect of preventive medicine which presents itself to the general practitioner is somewhat different, and deserves to be emphasised. Preventive medicine as applied to the social body is one thing, and as applied to the individual body is another. It is this latter which in all his work should be the guiding principle of the general practitioner. Furthermore, one must distinguish between absolute prevention and relative prevention. The practical applications of the latter will be found to be the more numerous. By absolute prevention is to be understood the anticipating and turning aside of some particular disease. From the definition already given of the meaning of disease it will be realised how absolute is the dependence of prevention upon knowledge of causation. The practitioner cannot give advice which will lead to a patient avoiding an attack of psoriasis, urticaria, asthma, migraine, rheumatoid arthritis, or glaucoma, unless he is aware of the cause of those conditions. On the other hand, dimness of sight due to tobacco, alcoholic cirrhosis of the liver, and all the disastrous and far-reaching results of venereal infection, can be anticipated and turned aside. Relative prevention will present itself as a practical problem in the daily work of every physician. In the contemplation of established, incurable, and even fatal disease, the question must arise in every thoughtful mind, Was there not once a stage in this process when something might have been done? was there not a time when this patient stood at the parting of the ways? and was there no sign-post to indicate the right road? The practitioner who seeks to do his work along preventive lines must ever bear in mind the words of the poet-philosopher: "Oh, small beginnings, ye are great and strong."

I have already dwelt on the impossibility of coping with the problem of prevention until we have first settled the question of causation. Etiology, the study of causes, should be, in accordance with the definition already given, a province of diagnosis. The only true diagnosis, it will be remembered, is the correlation of cause and effect in the individual patient. It may be useful to have in compendium form the presently recognised possible causes of disease. They may be classified under the following seven headings:—

1. First comes trauma. This will always be with us, so that we shall still have the need of the surgeon, even when all the other causes of ill-health have been "prevented" out of existence. For trauma, bringing in its train bruises and contusions, strains and sprains, wounds and all their infections, fractures, burns and scalds, frostbite and corrosive poisons, will provide work to his hand.

2. Next on the list is poisoning, including all the intoxications, whether of alcohol in "the cup that clears to-day of present ills and future fears" or of opium and its congeners in "the drowsy syrups" that cloud the mind and paralyse the body.

3. Parasitic causes of disease are both very numerous and of great variety, and include all the infections—typhoid fever, pneumonia, septicæmia, etc.

4. Heredity determines that, apart from all the accidents and incidents of environment, the individual comes into this world with a certain physical and mental endowment—it may be of but a few cubits, or of but one talent, so that these feeble ones are at best, and of necessity, continually on the border-line of disease.

5. Metabolic. Such conditions as gout and diabetes, and the disorders of the endocrine glands will find themselves in this group, at any rate provisionally. I say "provisionally" because we really need to know, in hyper-thyroidism for example, what is the *causa causans* of that condition.

6. Neoplastic causes represent all the new growths, both simple and malign. Further research may show that these are in part parasitic, in part metabolic; that is to say, due to invading microscopic enemies, or to a social revolution in the republic of body-cells.

7. Moral causes of disease make the seventh and last group. Here we trespass upon the sacred cult of psycho-analysis, for the brunt of action of moral causes must of necessity fall upon the higher functions of the nervous system. Our knowledge of mental processes and mental disease seems to be advancing along two parallel lines, which may be produced indefinitely but will never meet. The older method, and one which is in danger of being over-shadowed and overlooked, is that of working out the connection between psychosis and neurosis, the association, that is, of mental activity with nervous change, and the identification of function with certain definite areas of brain-tissue or groups of nerve-cells. The newer method of psycho-analysis endeavours to unravel the mental complexes without reference to territorial limitations. Its greatest danger at the present moment is the dominance of the sex-idea, which assumes that a man's whole nature revolves on a pivot of sexual feeling, and that the dissatisfactions and repressions of erotic experience account for all the psychic morbidities. Some investigators seem to be threatened with submersion in this *cloaca maxima* of sexual obsession.

Our knowledge of the cause and prevention of the mental and moral group of diseases seems somewhat to lag behind. It will not advance until we have regained the tradition, handed down to us from the Greek Ἰατρός, of the priestly as well as the healing function of the physician, and until we have learned to see and deal with man's nature in its perfect whole of body, soul, and spirit.

But little time remains to me in which to say anything of surgery, or cheirurgery, to use the older term which is preserved, fossilised as it were in the rock of our Society—a term which carries with it the essential idea of handicraft. But craftsmanship is not the whole of surgery, and it is not until, as in the name of our Society, medicine and cheirurgery are conjoined in matrimony that the latter can be fruitful. The surgeon must be a physician first, one who studies physics, that is Nature; but the physician need not be a surgeon. He may stop short of the exercise of that particular form of the craft. It is the study of the processes of disease which is common ground with both. At the marvellous advances which surgery has made, the extraordinary developments of operative technique, and the new fields in which she has won victories, one can only stand and look on with a sort of awed wonder. But these very advances carry with them an insidious danger to surgeons themselves. The ease and impunity with which even major operations can be undertaken tend to displace from their rightful position the less showy and more laborious methods of clinical investigation. Why waste time over inspection, palpation, mensuration, when it is so much easier to cut down and look? If the first incision is in the wrong place he just makes another, and they both heal by first intention. The instruments of precision, too, threaten to oust those other instruments, by no means unprecise, with which Nature has endowed us. "The eye first and most, the hand next and less, the tongue last and least." The marvellous accuracy of the older generation of surgeons in the employment of the unaided senses fills one with both wonder and envy. The eyes of a man like Sir Astley Cooper, for instance, seem to have been like an X-ray apparatus in miniature. No surgeon can pursue his craft with ability and success without some knowledge of anatomy, but that knowledge need not be of the exceedingly minute and detailed character which is such a feature of latter-day anatomical teaching. Indeed, anatomical teaching of a certain sort is a positive hindrance to the surgeon, who requires to be acquainted only with the principal landmarks of the country through which he is travelling, and he can pass by unnoticed the leaves on the hedgerows. Let the anatomist of the future, then, teach his students with less attention to detail, and more with regard to broad surgical principles.

Knowledge of First Aid comes painfully later still to embarrass the budding practitioner, who has only been taught how to deal with surgical emergencies when surrounded with all the resources and apparatus of a well-equipped institution.

In conclusion, lest any be discouraged by the contemplation of the long gap which still separates us from perfect knowledge and perfect skill, let us remember that wise and true saying: "La Médécinè guérit quelquefois, soulage souvent, console toujours."

